# Futile Care; Concept Analysis Based on a Hybrid Model

**DOI:** 10.5539/gjhs.v6n5p301

**Published:** 2014-08-15

**Authors:** Fatemeh Bahramnezhad, Mohammad Ali Cheraghi, Mahvash Salsali, Parvaneh Asgari, Fatemeh Khoshnavay Fomani, Mahnaz Sanjari, Pouya Farokhnezhad Afshar

**Affiliations:** 1 School of Nursing and Midwifery, Tehran University of Medical Sciences, Tehran, Iran; 2 Center for Nursing Care Research, School of Nursing and Midwifery, Tehran University of Medical Sciences, Tehran, Iran; 3 Nursing and Midwifery Faculty, Arak University of Medical Sciences, Arak, Iran; 4 Gerontology Department, Nursing and Midwifery Faculty, University of Social Welfare and Rehabilitation, Tehran, Iran

**Keywords:** futile care, nursing, concept analysis, Hybrid model

## Abstract

**Background:**

Making decision about what kind of caring is entitled as futile care requires the presentation of a clear definition of such caretaking.

**Objective:**

To report an analysis of the concept of futile care.

**Design:**

The analysis in this research was carried out through hybrid model in three stages. At the theoretical stage: a review of the available literature. At the work-in-field stage: semi-structured interviews.

**Setting:**

Data collection was on cancer unit and palliative care unit.

**Participants:**

A total of 7 participants were recruited in the study. The inclusion criteria were: having at least a bachelor’s degree in nursing, having at least 5 years of experience in critical care or cancer units, and being willing to participate in the study.

**Results:**

Three themes emerged: “low quality of life”, “lack physiologic return to life” and “performing non-professional duties”.

**Conclusion:**

Futile care consists giving clinical cares irrelevant to a nurse’s job and giving cares through which the return of patient would be impossible both physiologically and qualitatively.

## 1. Objective

Clinical decision making includes unique inter-patient transactions or even between their families and doctors. Ideally, patients or their families specify their own clinical cares results or intended objectives. Then, the doctor decides whether these decisions are operational or attainable and how much economical or human expenditures are incurred.

Nowadays, the demand for ICU bed has been increased due to the growth in the elderly population and the development of new therapeutic methods for life-threatening chronic diseases which implicate in certain problems.

This issue has led to the necessity for expressing emergency discussion about futile care (Davila, 2006). Doing such cares not only incurs a lot of expenses on healthcare system but also leads to emotional engagement of families, patients and healthcare personnel (McCabe & Storm, 2008).

Reviewing the literature about the definition of futility in medicine has greatly increased in recent years so much so that diverse professional groups including medicine, law, and politics have participated to act in the realm of futile care (Sibbald et al., 2007). The most important problem in this regard is the non-specification of boarders of a care that could identify it as futile. This in turn is due to the lack of giving a unified and obvious definition of the concept. Something that is considered as futile care for a therapeutic team might not be interpreted the same for another team or in another city or country (Brody & Halevy, 1995). Discussing futility or lack of futility for a care, it must be made clear what is meant by futility or lack of it in this realm and culture. There have been a lot of questions in medicine and medical ethics about this issue and a series of divers argumentations have been expressed (Palda et al., 2005). Due to different roles of doctors and nurses, their perception of the nature and manifestations of futile care for end-of-life patients are extremely different. The gap between the perceptions of healthcare providers is due to the lack of equality in judging and defining the realistic aims for therapy and intervention from the side of caretaking team (Kopelman, 1995). The current discussions over futile care have confronted individuals with a sort of confusion. Greener believes that the profuse number of superficial vague about the definition of futile care has led to the constriction of the usefulness of this expression and its usage in clinical fields (Griener, 1995). However, the presence of problems like attending to the independence right of the patient mentioned in the law and ethics are among the most important challenges caused within the field of futile care. Some believe that judging futility is among the privileges that are within the prowess of medical profession which has more value than the patient’s individual independence (Meltzer & Huckabay, 2004). Also stating, futile care is not affiliated with the country’s religious and cultural context. For example, Muslims believe that human life is in God’s hands until the last moment to try to save lives. The DNR order isn’t permitted (Peymani et al., 2012). Iranian culture due to high emotions and guilt of the last moment of life is a sick person trying to keep him alive (Farahanchi et al., 2012). One of the most important points that have been mentioned regarding futile care is the lack of a satisfactory and clear definition of it. Hence, presenting a true definition for the concept can be determined in giving care to patients and the manner of caring. This study has been carried out with the aim of reaching a clearer and more vivid definition for the concept of futility and its features.

## 2. Design

The hybrid model helps to clarify, identify, analyze and refine concepts at preliminary phases of developing a theory. The model has the highest usage rate in nursing (Rodgers & Knafl, 2000). The method relies on concept development and is executed through qualitative explorations of a phenomenon in its place of occurrence. In this method, a certain approach is adopted in which the theoretical and experimental methods are merged with each other and the final development of the concept is attained through induction and comparison so that a form of reduction is formed at the end (Seomun et al., 2006).

Concept analysis was done according to the hybrid model in three phases: the theoretical phase, the fieldwork phase and the analytical phase (Oh & Kang, 2005). In the theoretical phase, a literature review was conducted by getting help from credible databases like CINHAL, MEDLIN, Web of Science, Google Scholar, Ovid, IranMedex, and MagIran alongside the Oxford Dictionary of Medical Terms. The search key words were: inefficiency care, futile care, futility, and inappropriate care. The search covered the years from 1992 to 2012. A total of 119 English papers and one Persian paper were found, among them, 30 papers met the inclusion criteria of this study. At the fieldwork phase, the semi-structured interviews were done with 7 nurses.

## 3. Result

### 3.1 Phase 1: The Theoretical Phase

We started our search about futile care within the literature with these questions: What is the nature of futile care? How has futile care been defined? How is a futile care conceptualized? How is a futile care measured?

Most of the papers found at the theoretical phase had been in fact reviews of the definition and conceptualization of futile care (Hutchfield, 1999; RajPersaud et al., 2006; Davila, 2006); some of these papers had dealt with these concepts through mentioning examples like cardiopulmonary resuscitation of end-of-life patient, artificial respiratory supports by using ventilator machine when facing with failures in some organs, using multifarious antibiotics for the end-phase patient at his/her final moments of life and issues like these (Table l) (Griener, 1995; Tomlinson et al., 2001).

**Table 1:** The definitions observed in the theoretical phase

Source	Measurement	Defining the concept of futile care	Example
Davila, 2006	Moral Distress Scale (MDS)	Absence of a clear definition of futile care.	
McCabe and Storm, 2008	Literature review	Clinical cares have been quantitatively defined and it has been construed that when the survival chance is less than 5 per cent, giving care would be futile.
		- Inability or ineffectiveness happens with regard to achieving the intended goal; in this condition, remaining alive solely might not be enough.
		Futility has also been defined in spoken language as the ambiguity involved in making definite interventions that are meaningless and useless. This term, in medical literature, indicates the ambiguity involved in identifying the ineffective interventions and the totality of the term as well as the feeling of failure in giving futile care.
		- Futility is the ineffectiveness in achieving special purposes. If the intended objective is not fulfilled or the intended objectives cannot be achieved, caring would be futile.	
Sibbald et al., 2007	Literature review	Is used in intensive care units for patients who are at the end-of-life phase.	
Brody and Halevy, 1995	Literature review	- The pursuit of caring for an individual who seems to have no significant chance of survival.	
Palda et al., 2005	Corely Moral Distress Scale (CMDS)	Doing an action that rules out the possibility of achieving the objective	
Kopelman, 1995	Literature review	An intervention that does not benefit an individual sign is divided into two groups of futility: physiologic and qualitative.	Cardio pulmonary resuscitation of a patient whose chance of survival is very low. Resuscitation a patient with persistent vegetative state.
Griener, 1995	Literature review	When an intervention does not slow the progression signs of a disease or does not reduce its symptoms or even results in decreasing the patient’s quality of life.	Intravenous Infusion, therapeutic antibiotic, the onset of the second phase of chemotherapy for a patient who is at the end phase, starting respiratory supports for end-phase patient.
Meltzer and Huckabay, 2004	Moral Distress Scale (MDS) Malach Burnout Inventory (MBI)	An action is futile whose objective is not attainable or its success is improbable regarding past experiences. Extensive and aimless care in intensive care units.	Providing CPR for a patient with deficiencies in different limbs.
Peymani et al., 2012	Interview semi structure	Noticeable use of resources is one reason for having hope in the recuperation of the patient or when s/he makes a partial independence or interaction with the environment. Pain and torment are not necessary in defining a situation as futile but they might be examples of futile care for the team which provides caring and hence can be considered as annoying.	Support through the ventilator.
Farahanchi et al., 2012	Literature review	Physiologic futility: Is defined as lack of giving intervention to physical targets; if resuscitation does not have the ability of restoring the blood flow to a normal state and is practically futile, then it indicates futility.	Cardiopulmonary resuscitation for a patient with vegetation life.
		Qualitative futility: It encompasses the cases in which the desired conditions for reaching to a goal are challenged despite the possibility for the treatment to be effective. Futility is providing care for somebody whose rate of treatment chance is very low due to his/her low quality of life.	- Mechanical ventilation support of a patient who has only a few days of survival.
Rodgers and Knafl, 2000	Electronic trauma registry data base patient medical record	Resuscitating someone who has been taken to hospital while suffering from arrest and whose chance of resuscitation is very low.	

The Medical Terms Dictionary has defined futile care as a set of interventions that do not lead to improvement in the patient’s pre-awareness, relief, comfort, and the therapeutic-hygienic condition.

According to the literature review, an operational definition of futile care is as providing clinical services (diagnostic, preventive, therapeutic, and rehabilitative) for a patient whose possibility of returning to life both physiologically and qualitatively is less than 5 per cent.

In reviewing the literature on futile care, lack of fulfillment in goal by doctors in reviving a patient has also been defined. According to our literature review, Council on Ethical and Judicial Affairs first proposed concept of futile care in 1999 (Council on Ethical and Judicial Affairs, 1999).

### 3.2 Phase 2: The Fieldwork Phase

#### 3.2.1 Theoretical Framework

The researchers used the theoretical framework for guiding the interviews at the fieldwork phase. The first author directed all the interviews with asking open-ended questions and semi-structured interviews. The questions used in semi-structured interviews were designed by reviewing the literature and further questions were derived from the participants’ responses. For instance, some of the questions were as follows:

What kind of feeling did you have to give care to a patient whose alertness level was low and you knew that s/he would not survive after giving all medical services? Why?

Why did you give care to the patient despite knowing that s/he would not survive? Why do you resuscitate him/her? What is your definition of caring for these patients?

#### 3.2.2 Sampling

Sampling from the targeted group started following the receipt of confirmation from the Ethics Committee. The inclusion criteria were: having at least a bachelor’s degree in nursing, having at least 5 years of experience in critical care or cancer units, and being willing to take part in the study. A total of 7 participants entered the study and their demographic data is presented in Table 2.

**Table 2:** Participants’ demographic data

Demographic specifications	Frequency
Gender	4 females/3 male
Educational degree	5 bachelor’s and 2 master’s degree
Average age	46.50 years
Average time of work at intensive care unit	12-30 years

#### 3.2.3 Approach

The interviews were conducted face to face and took at least 35 to 50 minutes. Whenever a problem was observed during the interviews, the researchers would contact the participant by phone and talk to him/her for 10 to 15 minutes. The place in which the interview was conducted depended on the participants’ preference; one of them liked to be interviewed in the researcher’s office so the interview was carried out at the researcher’s office at the Department of Nursing and Midwifery in Tehran.

The other participants were willing to do the interviews in the evening shift at their own workplace. After getting oral and written informed consent, the interviewee entered the interview. All interviews were recorded and transcribed verbatim by the first author. Following the transcription of the interviews, their texts were given to the participants again and they all approved of whatever they had said and was accordingly transcribed by the researcher. For instance, some of the questions were as follows:

What kind of feeling did you have to give care to a patient whose alertness level was low and you knew that s/he would not survive after giving all medical services? Why?

Why did you give care to the patient despite knowing that s/he would not survive? Why do you resuscitate him/her? What is your definition of caring for these patients?

### 3.3 Data Analysis

All the collected data from the interviews were analyzed through qualitative approach and content analysis. After transcribing the interviews verbatim, they were coded. At first, 200 initial codes were extracted and through reductive classification, the categories and subcategories emerged. Following the content analysis, 10 codes emerged. In order to ensure the reliability of coding, the first author manually categorized the data at first and then they were reviewed by the second author. After finishing the data analysis, the researchers compared all the themes emerging from the interview data with the themes created through literature review and the similarities and differences between the emerged themes and those obtained through literature review were jotted down.

Theme 1: Low quality of life

One of the dimensions of futile care, which was frequently mentioned by the participants, was caring for a patient whose quality of life was low. The participants admitted that therapeutic and caring-related expenses for individuals who would require medical equipment’s and caring despite survival are considered as futile care.

“*Imagine a patient whose C3 and C4 is broken and is also suffering from quadriplegia and kidney loss; if you are supposed to regularly give CPR to him/her, what is the advantage? If s/he returns to life, s/he would require to be attached to machinery and get caring for the rest of her/his life which makes it counterproductive for both themselves and their families*.”

Theme 2: Lack of physiologic return

This theme indicated that the participants considered it as futile care if it is medically proven that a patient is unable to survive despite getting medical care.

“*We had a patient who received 40 drops of adrenaline but still suffered from a hypotension; if the adrenaline injection was stopped, he would have survived up to 24 hours which could compel us then to provide TPN.”*

Theme 3: Doing non-professional duties

The third theme obtained from coding the interviews and was in contrast with what was observed in literature review was doing non-professional duties. All 7 participants in the interviews mentioned that if they ever did anything else other than direct and indirect caring for a patient, it would be construed as futile caring. They also admitted that due to this fact that nurses do not have a clear checklist of duties, they might do some actions that could be useless for the patient and accordingly could be construed as futile care; in other words, a nurse would opt to do these actions despite having no use for the patients.

“ Sometimes, I think to myself that some of my actions at the hospital are like beating my head against a brick wall; I am forced to deliver tranquilizers or even do some actions that are within the duty domain of the ward secretary; hence, I am left behind my main responsibilities.”

### 3.3 Phase III: The Analysis Phase

Regarding the definition of futile care we came across 3 main themes among which 2 were in accordance with those resulting from the literature review. Giving care to a patient with low quality of life was the common theme with the literature review. Aramesh (2010) believes that futile care occurs when the quality of intervention is very weak and useless (Aramesh, 2011). Futile care includes a series of clinical conditions in which the explanations by doctors and therapeutic team remained in a way that such caring does not assist treatment and will not result in further comfort for the patient, does not take the quality of care back and does not result in patient’s consent (Rivin, 1997). In addition, individual’s lack of physiologic return to life was another main theme that was the same as the one obtained through the literature review in theoretical phase. Neiderman and Berger (2010) believe that futile care is attributed to a kind of care that does not have any chance for achieving acceptable results. Futile care happens when we even achieve the desired result of an intervention but the question would be over its usefulness. Consequently, the discussion over futile care is formed about the probable usefulness of an intervention (Niederman & Berger, 2010).

In contrast with the findings of the literature review, one main theme emerged out of our analysis following the interviews with participants; “doing non-professional duties”. The researcher thinks that due to the emergence of this theme out of the interviews, it could be deemed as context based. Moreover, this theme might have emerged by virtue of the fact about the lack of a clear checklist of duties for nurses or lack of awareness despite its existence, which was admitted by participants. The consequences of giving futile care enjoyed a high interaction between what had been obtained out of literature review and the fieldwork phase. Imposing expenditure (both on system and families), nurse’s job exhaustion and harming all patients were among the consequences of the futile care. The new theme in this phase was “patient’s psychological torment”. In the fieldwork phase, the participants believed that because of being a Muslim and believing in the unity of the soul and the body after death, giving futile care would result in tormenting patients’ souls. The reasons for giving futile care which were indicative of the interaction between the theoretical phase and the fieldwork phase were: ethical issues, fear of legal pressures, doctor’s attitude, lack of proper relations between caregivers’ team; patient and families, cultural and religious issues, emotions, and lack of having clear instructions. Besides, two new themes emerged from interviewing the participants: 1). unclear pre-awareness and expecting a miracle, 2). Human errors.

The participants believed that lack of coordination between doctor’s diagnosis and nurse and doctors’ opting for trial and error sometimes lead to giving futile care.

This content analysis was done merely from the nurses’ perspectives. The researcher hopes that this concept could be investigated and analyzed in the future from the viewpoints of doctors, patients and their families as well.

## 4. Conclusion

Futile care is a concept affected by a host of factors including: cultural; ethical; and religious issues, lack of coordination within the therapeutic personnel, patient and families, lack of communal training, commitment of human errors, unclear pre-awareness of diseases, lack of clear instructions, and finally work conditions. By the same token, it includes giving clinical services that are irrelevant to nurse’s work and administering cares that could not physiologically and qualitatively result in the resuscitating patient. As a result, its main consequences are: incurring enormous expenditures, burnout, patients and families’ tormenting which would finally lead to dissatisfaction of nurses doing such caring. Carrying out futile care is exhausting and tormenting and affects patients as well as their families.
